# Alarmingly Rising Trends in Venous Thromboembolic Events and Respiratory Failure in Takotsubo Syndrome-Related Hospitalizations in the United States

**DOI:** 10.7759/cureus.10985

**Published:** 2020-10-16

**Authors:** Hee Kong Fong, Zainab J Gandhi, Faizan A Malik, Ankur Panchal, Sejal Savani, Rajkumar Doshi, Rupak Desai

**Affiliations:** 1 Cardiovascular Medicine, University of California Davis Medical Center, Sacramento, USA; 2 Internal Medicine, C.U. Shah Medical College, Surendranagar, IND; 3 Internal Medicine, Texas Tech University Health Sciences Center at Permian Basin, Odessa, USA; 4 Internal Medicine, University of Pittsburgh Medical Center, Pittsburgh, USA; 5 Public Health, New York University, New York City, USA; 6 Internal Medicine, University of Nevada, Reno School of Medicine, Reno, USA; 7 Cardiology, Atlanta Veterans Affairs Medical Center, Decatur, USA

**Keywords:** takotsubo syndrome, venous thromboembolism (vte), respiratory failure, stress induced cardiomyopathy

## Abstract

Background

There is very scarce data about the shifting landscape of complications like venous thromboembolic events (VTE) and respiratory failure in Takotsubo syndrome (TTS). We have assessed the rates and trends of these complications in (TTS)-related hospitalizations.

Methods

The National Inpatient Sample (2007-2014) was queried to identify adult hospitalizations for TTS and subsequent VTE and respiratory failure using the International Classification of Diseases, Ninth Revision, Clinical Modification (ICD-9 CM) codes. Trends were assessed using discharge weights and the linear-by-linear association test for the overall cohort and subgroups based on age, sex, and race.

Results

Of 156,506 admissions for TTS from 2007-2014, 3.5% (N=5,550) of admissions revealed VTE whereas 17.4% (N=27,252) of admissions revealed respiratory failure. There were significantly rising trends in VTE (from 2.2% to 4.2%) and respiratory failure (10% to 20.7%) with TTS (p<0.05) from 2007-2014. On subgroup analysis, all subgroups showed rising trends in VTE and respiratory failure. However, young (18-44 years), male patients admitted with TTS demonstrated a greater surge in VTE as compared to other groups. In contrast, the frequency of respiratory failure rose more significantly in young, male, non-white TTS patients compared to older, female and white TTS patients.

Conclusion

There were alarming trends in the VTE and respiratory failure despite the improved understanding of TTS etiopathogenesis and advanced diagnostic modalities among TTS-related admissions, mostly comprising of young, male, and non-white patients. Introduction

## Introduction

The clinical manifestation of Takotsubo syndrome (TTS) or stress-induced cardiomyopathy closely resembles that of acute myocardial infarction; however, the most common triggers are either physical or emotional stressors [1]. While the exact pathophysiological mechanism of TTS remains to be established, various mechanisms have been suggested in the literature. One of the studies suggested the possible role of hyper-viscosity, which can induce physical stress or a transitory thrombotic occlusion of the coronary arteries [2]. Another study found higher serum levels of a von-Willebrand factor (VWF) and plasminogen activation inhibitor-1 (PAI-1) in TTS patients as compared to controls which further suggests the role of endothelial disorders in the pathogenesis of TTS [3]. The presence of higher levels of lipoprotein (a) in TTS patients when compared to controls favours hyper-viscosity rather than a prothrombotic state which can contribute to a higher propensity in developing TTS [4]. TTS patients can present with respiratory failure secondary to acute pulmonary edema from acute decompensated heart failure or exacerbation of underlying chronic pulmonary conditions [5]. Previous studies have discussed cardiovascular manifestations of TTS; however, large-scale data on the epidemiology of venous thromboembolism (VTE) and acute respiratory failure in TTS remains mostly obscure. This study aims to assess the frequency and trends of VTE and respiratory failure in TTS-related hospitalizations in the United States using a nationally representative sample.

## Materials and methods

We queried the National Inpatient Sample (NIS) from 2007 to 2014 with the International Classification of Diseases Clinical Modification, 9th Revision codes of TTS (429.83), VTE (453.40), and respiratory failure (518.51) to study the trends in TTS-related hospitalization (n=156,506). The NIS is a large, publicly accessible all-payer inpatient database in the United States, comprising data on nearly seven million hospital stays annually [6]. The NIS represents a stratified sample of 20% nonfederal US community hospitals which is demonstrative of nearly 95% of the United States population. The weighted NIS dataset provides nationwide estimates of over 35 million hospitalizations annually. The NIS contains patient-level data points, up to 25 discharge diagnoses up to 15 procedural diagnoses, and hospitalization details such as bed size, location/teaching status, and geographical region of hospitals. Since the NIS is a deidentified administrative database, institutional board approval was not mandatory.

The primary outcome was frequency and trends (overall and stratified by age, sex, and race) in VTE and respiratory failure in TTS-related admissions from 2007 to 2014. A linear by linear association test was used to assess trends. SPSS v24 (IBM Corp, Armonk, NY, USA) was utilized to perform statistical analyses using defined strata/cluster designs and weighted datasets.

## Results

The Demographics and outcomes of TTS-related hospitalization with VTE and respiratory failure were analyzed. The prevalence of VTE in TTS-related hospitalization was 3.5% (n=5,550), and the prevalence of respiratory failure in TTS-related hospitalization was 17.4% (n=27,252). The frequency of VTE (from 2.0% to 4.2%) and acute respiratory failure (10% to 20.7%) in TTS nearly doubled between 2007 and 2014 (ptrend<0.001).

**Figure 1:**
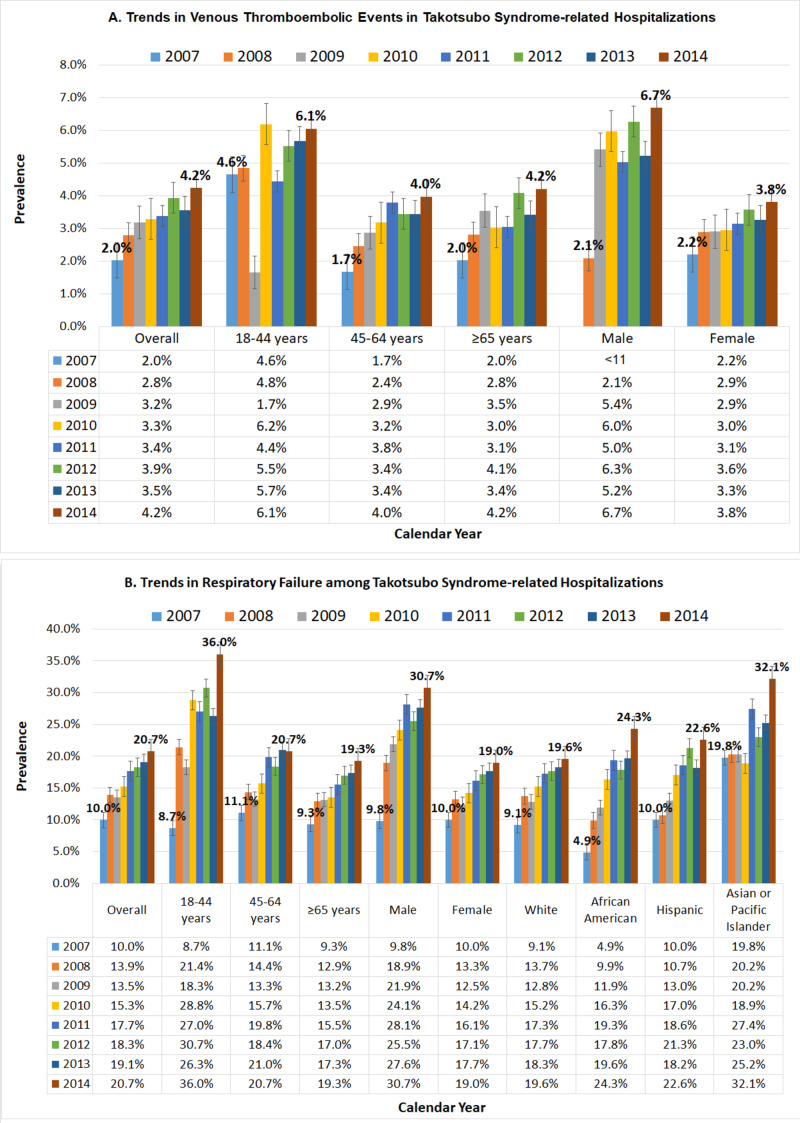
Trends in a) venous thromboembolic events and b) acute respiratory failure among Takotsubo syndrome-related hospitalizations from 2007 to 2014

On a further subgroup trend analysis, the frequency of VTE and respiratory failure in TTS-related hospitalization was higher in males, trends of which have increased from 2007 to 2014 (6.7% vs 3.8%). Rising trends in VTE and acute respiratory were seen from 2007 to 2014 in all age groups; however, most pronounced rising trends were observed in the younger age group of 18-44 years (8.7% to 36%, ~4 fold increase) (Figure 1).

## Discussion

In our analysis, we found more pronounced rising trends of TTS-associated VTE and acute respiratory failure in non-white populations with the African American population showing the highest increase (~5-fold increase) between 2007 and 2014. Khera et al. studied the differences in gender, race, and age for patients of TTS. Men with TTS were younger, often non-white, more likely to be black, and were associated with worse prognosis and higher rates of complications compared to women [7]. Men have shown to have higher in-hospital mortality and most commonly due to respiratory failure and cardiogenic shock [8]. The TTS-related hospitalization with VTE and respiratory failure demographically favour younger, non-white, male patients.

Our large study sample size suggests that VTE and respiratory failure in TTS-related hospitalizations should not be taken lightly, and we need to anticipate the development of these complications in TTS patients. Further studies are needed to understand the etiopathophysiology of VTE and respiratory failure in TTS-related hospitalizations. With the high prevalence of VTE and ultimately respiratory failure in TTS patients, anticoagulation should be considered in high-risk patients, independent of the fact that patients with apical ballooning patterns might benefit from systemic anticoagulation to prevent left ventricular thrombus formation which could ultimately lead to stroke [9,10]. However, the efficacy of such therapy in preventing worse outcomes in TTS patients needs to be further explored with randomized control trials.

There are some potential limitations in our study which include billing and coding issues possibly due to administrative data collection, lack of medication history, lack of longitudinal follow-up, potential comorbidities that have not been considered, and non-availability of laboratory results. Furthermore, there could be a possibility of residual confounders in this retrospective analysis which can be controlled with comprehensive multivariable analyses in future longitudinal studies. Despite these limitations, nationally representative cohorts of VTE, respiratory failure, and TTS enabled us to perform this analysis on one of the understudied subjects. The abstract of this article has been presented at the 'American College of Cardiology Conference' in March 2020 [11].

## Conclusions

Despite the improved understanding of TTS etiopathogenesis and the development of advanced diagnostic modalities over the last decade, there have been a rising trend in VTE and respiratory failure among TTS-related admissions from 2007 to 2014, especially comprising of young, male, and non-white patients. There is a dire need for further investigation into this trend to prevent life-threatening complications in TTS patients.
